# Chemotherapeutic drugs for soft tissue sarcomas: a review

**DOI:** 10.3389/fphar.2023.1199292

**Published:** 2023-08-11

**Authors:** Zhichao Tian, Weitao Yao

**Affiliations:** Department of Orthopedics, The Affiliated Cancer Hospital of Zhengzhou University and Henan Cancer Hospital, Zhengzhou, China

**Keywords:** chemotherapeutic drugs, sarcoma, review, chemotherapy, efficacy

## Abstract

Despite the low incidence of soft tissue sarcomas (STSs), hundreds of thousands of new STS cases are diagnosed annually worldwide, and approximately half of them eventually progress to advanced stages. Currently, chemotherapy is the first-line treatment for advanced STSs. There are difficulties in selecting appropriate drugs for multiline chemotherapy, or for combination treatment of different STS histological subtypes. In this study, we first comprehensively reviewed the efficacy of various chemotherapeutic drugs in the treatment of STSs, and then described the current status of sensitive drugs for different STS subtypes. anthracyclines are the most important systemic treatment for advanced STSs. Ifosfamide, trabectedin, gemcitabine, taxanes, dacarbazine, and eribulin exhibit certain activities in STSs. Vinca alkaloid agents (vindesine, vinblastine, vinorelbine, vincristine) have important therapeutic effects in specific STS subtypes, such as rhabdomyosarcoma and Ewing sarcoma family tumors, whereas their activity in other subtypes is weak. Other chemotherapeutic drugs (methotrexate, cisplatin, etoposide, pemetrexed) have weak efficacy in STSs and are rarely used. It is necessary to select specific second- or above-line chemotherapeutic drugs depending on the histological subtype. This review aims to provide a reference for the selection of chemotherapeutic drugs for multi-line therapy for patients with advanced STSs who have an increasingly long survival.

## 1 Introduction

Soft tissue sarcomas (STSs) are rare malignancies, accounting for only approximately 1% of all malignancies (Bhatt et al., 2016; Yang et al., 2019). There are more than 70 histological subtypes, and the clinical characteristics and prognoses of these subtypes greatly vary (Amadeo et al., 2020; Parikh et al., 2018; von Mehren et al., 2022; Tos et al., 2023). Despite the low incidence of STSs, hundreds of thousands of new STS cases are diagnosed annually worldwide, and approximately half of them eventually progress to advanced stages (Corey et al., 2014; Bhatt et al., 2016; Hung et al., 2019; Yang et al., 2019). Currently, chemotherapy is the first-line treatment for advanced STSs (Cojocaru et al., 2022; de Juan Ferre et al., 2021; von Mehren et al., 2020). Anthracyclines (mainly doxorubicin) were found to be effective against STSs in 1973 (Tan et al., 1973; Sritharan and Sivalingam, 2021). Since then, various clinical trials have been conducted to prolong survival or reduce adverse events in patients with STSs using intensive chemotherapy, non-anthracycline regimens, or alternative anthracycline drugs. These drugs include, but are not limited to, oxazaphosphorines, trabectedin, gemcitabine, taxanes, dacarbazine, eribulin, vinca alkaloid agents (vindesine, vinblastine, vinorelbine, vincristine), methotrexate, cisplatin, etoposide, and pemetrexed (Ratan and Patel, 2016; Hatcher et al., 2017; Smrke et al., 2020). The characteristics, efficacy, and safety of these drugs for STSs vary, and the responses of different STS subtypes to these chemotherapeutic drugs greatly vary. Although some drugs have shown good efficacy in individual subtypes, none have exceeded the efficacy and safety achieved by doxorubicin for STSs. To date, doxorubicin remains the first-line chemotherapeutic drug for STSs (Cojocaru et al., 2022; de Juan Ferre et al., 2021; von Mehren et al., 2020; Smrke et al., 2020; Smolle et al., 2020; Meyer and Seetharam, 2019; Yang et al., 2022; Gronchi et al., 2017). Selecting second- or higher-line drugs for advanced STS remains a challenge (Frezza et al., 2017; Kim et al., 2019; Haddox and Riedel, 2020; Younger et al., 2021; Kojima et al., 2022).

In the past decade, anti-vascular endothelial factor receptor multi-target tyrosine kinase inhibitors (TKIs), such as pazopanib, have been widely used in STS, which is a major breakthrough in the treatment of this type of malignancy, leading to significantly prolonged survival in patients with STS (Tang et al., 2021; Kyriazoglou et al., 2022; Thirasastr et al., 2022). Immunotherapeutic agents, such as programmed cell death protein 1 inhibitors, have also shown some therapeutic effects in some STS histological subtypes (Tang et al., 2021; Banks and D'Angelo, 2022; Tawbi et al., 2017). Furthermore, the combination of conventional chemotherapeutic drugs with targeted agents (TKIs or immunotherapeutic agents) is considered the next breakthrough in STS treatment (Tang et al., 2021; Patel et al., 2022; Principe et al., 2022; Tian and Yao, 2022). There are significant differences in the efficacy of different chemotherapeutic drugs combined with different targeted agents (Kyriazoglou et al., 2022; Principe et al., 2022; Fuchs et al., 2023). Based on the different STS subtypes, selecting potential chemotherapeutic drugs to combine with targeted drugs is important to achieve better efficacy (Principe et al., 2022; Tian and Yao, 2022). In recent years, few studies have systematically summarized the differences between the chemotherapeutic drugs used to treat STSs and the differences in the efficacy of these drugs in different STS subtypes. This leads to difficulties in selecting appropriate chemotherapeutic drugs for combination treatment of different STS subtypes.

In this study, we first comprehensively reviewed the efficacy of various chemotherapeutic drugs in the treatment of STSs, and then described the current status of sensitive drugs for different STS subtypes. We aim to provide a reference for the selection of chemotherapeutic drugs for multi-line therapy for patients with advanced STSs who have an increasingly long-survival.

## 2 Efficacy of different drugs in STSs

As a traditional method of cancer treatment, chemotherapy has been used in STSs for more than 50 years since the introduction of doxorubicin in the 1970s. Currently, chemotherapeutic drugs, such as anthracyclines, ifosfamide, trabectedin, gemcitabine, paclitaxel, dacarbazine, and eribulin, have therapeutic effects in STSs and are widely used in clinical treatment (Seddon, 2016; Bleloch et al., 2017; Frezza et al., 2017; Hatcher et al., 2017; Smrke et al., 2020). Here, we comprehensively reviewed the clinical trial results of various drugs for STSs to accurately describe the efficacy of them in STSs. To improve the reliability of this study, we attempted to use data from multicenter, prospective, phase II–IV clinical trials as much as possible. In the case of the absence of prospective clinical trial results, a multicenter retrospective study with large sample size conducted by multinational sarcoma organizations was included in the analysis. During our review, we found that the outcomes of different studies were presented using different measures, including the objective response rate (ORR), disease control rate, median progression-free survival (PFS), PFS rate, median overall survival (OS), and OS rate. We uniformly selected the most common measures, ORR, median PFS, and median OS, as comparative indicators.

### 2.1 Anthracyclines

Anthracyclines are among the most effective chemotherapeutics for cancer. They are glycoside drugs comprising the amino sugar daunosamine linked to a hydroxyanthraquinone aglycone, and they induce cell death through multiple intracellular targets: reactive oxygen species generation, DNA-adduct formation, topoisomerase II inhibition, histone eviction, Ca^2^ + and iron hemostasis regulation, and ceramide overproduction (Rabbani et al., 2005; Jasra and Anampa, 2018; Martins-Teixeira and Carvalho, 2020). Doxorubicin is the most effective and widely used anthracycline for the treatment of STSs (Table 1). Several other anthracyclines, such as aldoxorubicin, epirubicin, and pegylated liposomal doxorubicin, have also been used for the clinical treatment of STSs (Table 1). However, none of the other anthracyclines exceed the efficacy of doxorubicin in STSs (Table 1) (Nielsen et al., 2000a; Judson et al., 2001; Chamberlain et al., 2019; Martins-Teixeira and Carvalho, 2020; Peter et al., 2022).

**TABLE 1:** Outcomes of representative clinical trials of anthracyclines in nonspecific STSs.

Drugs and dosages	Years of report	Study types	Setting	Number of patients	Outcomes			References
					ORR (%)	M-PFS (months)	M-OS (months)	
**Single agent**								
Doxorubicin 75 mg/m^2^/d1/3w	2020	Phase III trial	Anthracycline-naive	251	18.3	6.8	19.7	Tap et al. (2020)
Doxorubicin 60 mg/m^2^/d1/3w	2020	Phase II trial	First-line	40	7.7	4.3	9.8	Hartmann et al. (2020)
Doxorubicin 75 mg/m^2^/d1/3w	2020	Phase II trial	First-line	39	15.4	5.3	14.3	Grünwald et al. (2020)
Doxorubicin 75 mg/m^2^/d1/3w	2017	Phase III trial	First-line	323	18	6.0	19.0	Tap et al. (2017)
Doxorubicin 75 mg/m^2^/d1/3w	2017	Phase III trial	First-line	129	20	5.4	17.9	Seddon et al. (2017)
Doxorubicin 75 mg/m^2^/d1/3w	2016	Phase II trial	Anthracycline-naive	65	11.9	4.1	14.7	Tap et al. (2016)
Doxorubicin 75 mg/m^2^/d1/3w	2016	Phase III trial	First-line	221	19.9	5.2	16.9	Ryan et al. (2016)
Doxorubicin 75 mg/m^2^/d1/3w	2016	Phase II trial	First-line	59	17	5.5	13.7	Martin-Broto et al. (2016)
Doxorubicin 75 mg/m^2^/d1/3w	2015	Phase II trial	First-line	40	5	4.6	14.3	Chawla et al. (2015)
Doxorubicin 75 mg/m^2^/d1/3w	2014	Phase III trial	First-line	228	14	4.6	12.8	Judson et al. (2014)
Doxorubicin 75 mg/m^2^/d1/3w	2009	Phase II trial	First-line	64	23.4	6.5	-	Maurel et al. (2009)
Doxorubicin 75 mg/m^2^/d1/3w	2007	Phase III trial	First-line	110	11.8	2.5	12.0	Lorigan et al. (2007)
Doxorubicin 75 mg/m^2^/d1/3w	2001	Phase II trial	First-line	44	7	2.7	8.2	Judson et al. (2001)
Doxorubicin 75 mg/m^2^/d1/3w	1998	Phase III trial	First-line	104	14	3.7	10.5	Nielsen et al. (1998)
Doxorubicin 75 mg/m^2^/d1/3w	1995	Phase III trial	First-line	263	23.3	10.7	12.1	Santoro et al. (1995)
Doxorubicin 70 mg/m^2^/d1/3w	1990	Phase III trial	First-line	151	17	3	9.4	Borden et al. (1990)
Doxorubicin 70 mg/m^2^/d1/3w	1987	Phase III trial	First-line	83	25	3.5	9.6	Mouridsen et al. (1987)
Aldoxorubicin 350 mg/m^2^/d1/3w	2015	Phase II trial	First-line	83	23	8.3	15.8	Chawla et al. (2015)
Pegylated liposomal doxorubicin 50 mg/m^2^/d1/4w	2001	Phase II trial	First-line	50	10	2.2	10.7	Judson et al. (2001)
Epirubicin 150 mg/m^2^/d1/3w	1998	Phase III trial	First-line	106	15	3.3	11.0	Nielsen et al. (1998)
Epirubicin 50 mg/m^2^/d1–3/3w	1998	Phase III trial	First-line	106	14	2.8	10.5	Nielsen et al. (1998)
Epirubicin 75 mg/m^2^/d1/3w	1987	Phase III trial	First-line	84	18	2.8	11.2	Mouridsen et al. (1987)
**Combination regimens**								
Doxorubicin 75 mg/m^2^/d1/3w plus olaratumab 20 mg/kg/d1,8/3w in cycle 1, and 15 mg/kg in subsequent cycles	2020	Phase III trial	First-line	258	14	5.4	20.4	Tap et al. (2020)
Doxorubicin 75 mg/m^2^/d1/3w plus evofosfamide 300 mg/m^2^/d1, 8/3w	2017	Phase III trial	First-line	317	28	6.3	18.4	Tap et al. (2017)
Doxorubicin 75 mg/m^2^/d1/3w plus olaratumab 20 mg/kg/d1, 8/3w	2016	Phase II trial	First-line	65	18.2	6.6	16.5	Tap et al. (2016)
Doxorubicin 75 mg/m^2^/d1/3w plus palifosfamide 150 mg/m^2^/d1–3/3w	2016	Phase III trial	First-line	226	28.3	6.0	15.9	Ryan et al. (2016)
Doxorubicin 60 mg/m^2^/d1/3w plus trabectedin 1.1 mg/m^2^/d1/3w	2016	Phase II trial	First-line	54	17	5.7	13.3	Martin-Broto et al. (2016)
Doxorubicin 25 mg/m^2^/d1–3/3w plus ifosfamide 2.5 g/m^2/^d1-4/3w	2014	Phase III trial	First-line	228	26	7.4	14.3	Judson et al. (2014)
Doxorubicin 30 mg/m^2^/d1–3/2w for 3 cycles followed by ifosfamide 2.5 g/m^2^/d1-5/3w for 3 cycles	2009	Phase II trial	First-line	62	24.1	6.0	-	Maurel et al. (2009)
Doxorubicin 20 mg/m^2^/d1–3/3w plus ifosfamide 2.5 g/m^2^/d1–3/3w plus dacarbazine 300 mg/m^2^/d1–3/3w	2009	Phase III trial	First-line	74	35	9.8	17.7	Fayette et al. (2009)
Doxorubicin 25 mg/m^2^/d1–3/3w plus ifosfamide 3 g/m^2^/d1–3/3w plus dacarbazine 400 mg/m^2^/d1–3/3w	2009	Phase III trial	First-line	71	38	9.1	17.3	Fayette et al. (2009)
Doxorubicin 30 mg/m^2^/d1–3/2w for 3 cycles followed by ifosfamide 2.5 g/m^2^/d1–5/3w for 3 cycles	2004	Phase II trial	First-line	57	38	5.6	13.5	Maurel et al. (2004)
Pegylated liposomal doxorubicin 45 mg/m^2^/d1/3w plus paclitaxel 150 mg/m^2^/d1/3w	2004	Phase II trial	First-line	42	16	5.7	13.2	Bafaloukos et al. (2004)
Doxorubicin 50 mg/m^2^/d1/3w plus ifosfamide 5 g/m^2^/d1/3w	2000	Phase III trial	First-line	149	21	11.0	13.1	Le Cesne et al. (2000)
Doxorubicin 75 mg/m^2^/d1/3w plus ifosfamide 5 g/m^2^/d1/3w	2000	Phase III trial	First-line	145	23.3	8.6	12.8	Le Cesne et al. (2000)
Doxorubicin 50 mg/m^2^/d1/3w plus ifosfamide 5 g/m^2^/d1/3w	1995	Phase III trial	First-line	258	28.1	10.3	12.8	Santoro et al. (1995)
Cyclophosphamide 500 mg/m^2^/d1/3w plus vincristine 1.5 mg/m^2^/d1/3w plus doxorubicin 50 mg/m^2^/d1/3w plus dacarbazine 750 mg/m^2^/d1/3w	1995	Phase III trial	First-line	142	28.4	11.2	11.9	Santoro et al. (1995)
Doxorubicin 60 mg/m^2^/d1/3w plus dacarbazine 1,000 mg/m^2^/d1/3w	1993	Phase III trial	First-line	170	17	4	12	Antman et al. (1993)
Doxorubicin 60 mg/m^2^/d1/3w plus dacarbazine 1,000 mg/m^2^/d1/3w plus ifosfamide 5–7.5 g/m^2^/d1–3/3w	1993	Phase III trial	First-line	170	32	6	13	Antman et al. (1993)
Doxorubicin 70 mg/m^2^/d1/3w plus vindesine 3 mg/m^2^/d1/3w	1990	Phase III trial	First-line	147	18	4	9.9	Borden et al. (1990)
Doxorubicin 60 mg/m^2^/d1/3w plus ifosfamide 5 g/m^2^/d1/3w	1989	Phase II trial	First-line	42	36	7	8	Loehrer et al. (1989)
Doxorubicin 60 mg/m^2^/d1/3w plus dacarbazine 900 mg/m^2^/d1/3w plus ifosfamide 7.5 g/m^2^/d1/3w	1989	Phase II trial	First-line	105	47	9.5	16	Elias et al. (1989)
Doxorubicin 60 mg/m^2^/d1/3w plus dacarbazine 250 mg/m^2^/d1/3w	1987	Phase III trial	First-line	104	33	7.2	8.6	Baker et al. (1987)
Doxorubicin 60 mg/m^2^/d1/3w plus dacarbazine 250 mg/m^2^/d1/3w plus cyclophosphamide 500 mg/m^2^/d1/3w	1987	Phase III trial	First-line	112	34	6.0	9.8	Baker et al. (1987)
Doxorubicin 60 mg/m^2^/d1/3w plus dacarbazine 250 mg/m^2^/d1/3w plus actinomycin D 1.2 mg/m^2^/d1/3w	1987	Phase III trial	First-line	119	24	5.4	11.7	Baker et al. (1987)

STSs, soft tissue sarcomas; ORR, objective response rate; M-PFS, median progression-free survival time; M-OS, median overall survival.

Doxorubicin (adriamycin) was isolated from *Streptomyces suis* and *S. peucetius* in the late 1960s (Tan et al., 1973; Peter et al., 2022). Since its Food and Drug Administration approval in 1974, doxorubicin alone or in combination with other drugs has been widely used as a first-line therapy for a myriad of cancers (Aubel-Sadron and Londos-Gagliardi, 1984; Sun et al., 2017). Doxorubicin induces cell death through multiple intracellular targets, including reactive oxygen species generation, DNA adduct formation, topoisomerase II inhibition, histone eviction, Ca^2^ + and iron hemostasis regulation, and ceramide overproduction. Moreover, doxorubicin-treated dying cells undergo cellular modifications that enable neighboring dendritic cell activation and enhance the presentation of tumor antigens. In addition, doxorubicin aids in the immune-mediated clearance of tumor cells (Carvalho et al., 2009; Sritharan and Sivalingam, 2021).

To date, Numerous clinical trials have demonstrated the efficacy of doxorubicin alone or doxorubicin-based chemotherapy for the treatment of STSs (Sritharan and Sivalingam, 2021; Peter et al., 2022). The results of the representative multicenter prospective clinical trials over the past 30 years are listed in Table 1. Due to various reasons such as the long-time interval between different clinical trials and errors caused by small sample sizes, the reported efficacy of doxorubicin monotherapy for STS varies significantly. However, clinical trials with sample sizes exceeding 100 in the past decade have shown that the ORR of doxorubicin monotherapy with a conventional dose (75 mg/m^2^/d1/3w) for STSs was 14%–20%, with a median PFS of 4.6–6.8 months (Table 1).

To further improve the efficacy of chemotherapy, doxorubicin in combination with other drugs has also been widely used (Table 1). The drug most commonly used in combination with doxorubicin is ifosfamide. The ORR of doxorubicin plus ifosfamide in treating STSs is 21%–38%, with a median PFS of 5.6–11 months (Table 1). Although the combination of doxorubicin and ifosfamide improves the ORR and median PFS compared with doxorubicin alone, it does not improve the median OS (Table 1) and instead increases hematological toxicity such as leucopenia and anemia (Maurel et al., 2009; Judson et al., 2014; Wang et al., 2021). Therefore, recently, this combined regimen is not recommended as a first-line chemotherapy for advanced STSs but is only recommended for preoperative neoadjuvant chemotherapy of high-risk STSs (Judson et al., 2014; Weiss et al., 2020). Furthermore, no combination regimen has been shown to significantly extend the median OS over doxorubicin monotherapy in patients with advanced STSs (Table 1). Notably, the doxorubicin plus ifosfamide plus dacarbazine achieves the highest ORR (38%) and median PFS (9.8 months) in patients with advanced STSs (Table 1). This combined regimen should also be tested in the setting of neoadjuvant therapy.

In summary, as the most recognized chemotherapeutic drug, doxorubicin is the cornerstone of advanced STS chemotherapy. The testing of new drugs in the field of STSs is always guided by doxorubicin. With the invention and testing of an increasing number of targeted and immunotherapeutic drugs, various doxorubicin-based combination therapies will be widely tested and applied for the treatment of STSs.

### 2.2 Oxazaphosphorines

Oxazaphosphorines are a class of bifunctional alkylating agents that have been extensively investigated over the past 50 years and have a wide spectrum of anticancer and immune-regulating activities (Giraud et al., 2010). Most oxazaphosphorines are designed as prodrugs that require cytochrome P450 enzyme-mediated bioactivation to generate highly reactive alkylating nitrogen mustards, which exert their chemotherapeutic effects by attacking specific nucleophilic groups of DNA molecules in target cancer cells (Misiura, 2006; Liang et al., 2007; Wang and Wang, 2012). In STS chemotherapy, ifosfamide is the most widely used oxazaphophorine with definite efficacy (Table 2). Other oxazaphosphorines, such as cyclophosphamide, trofosfamide, evofosfamide, and palifosfamide, have also been used for the treatment of STSs (Table 2). However, to date, none of the other oxazaphosphorines have exceeded the efficacy of ifosfamide in STSs (Table 2) (Giraud et al., 2010; Mulder et al., 2015; Tap et al., 2017; Hartmann et al., 2020).

**TABLE 2:** Outcomes of representative clinical trials of oxazaphosphorines in nonspecific STSs.

Drugs and dosages	Years of report	Study types	Setting	Number of patients	Outcomes			References
					ORR (%)	M-PFS (months)	M-OS (months)	
**Single agent**								
Ifosfamide 3 g/m^2^/d1–3/3w	2007	Phase III trial	First-line	109	5.5	2.2	10.9	Lorigan et al. (2007)
Ifosfamide 9 g/m^2^/3 days continuous infusion/3w	2007	Phase III trial	First-line	107	8.4	3.0	10.9	Lorigan et al. (2007)
Ifosfamide 5 g/m^2^/d1/3w	2002	Phase II trial	1–2 line	49	10	2.6	12	van Oosterom et al. (2002)
Ifosfamide 3 g/m^2^/d1–3/3w	2002	Phase II trial	1–2 line	49	25	3.3	10	van Oosterom et al. (2002)
Ifosfamide 12 g/m^2^/3-day continuous infusion/4w	2000	Phase II trial	1–2 line	114	16	3.5	12.8	Nielsen et al. (2000b)
Ifosfamide 2 g/m^2^/d1–4/3w	1989	Phase II trial	First-line	110	24	-	7.2	Antman et al. (1989)
Ifosfamide 5 g/m^2^/d1/3w	1987	Phase II trial	First-line	68	18	-	-	Bramwell et al. (1987)
Trofosfamide 300 mg/d on days 1–7, then 150 mg/d continuously	2020	Phase II trial	First-line	80	6.6	2.8	12.3	Hartmann et al. (2020)
Cyclophosphamide 1.5 g/m^2^/d1/3w	1987	Phase II trial	First-line	67	8	-	-	Bramwell et al. (1987)
**Combination regimens**								
Doxorubicin 75 mg/m^2^/d1/3w plus evofosfamide 300 mg/m^2^/d1,8/3w	2017	Phase III trial	First-line	317	28	6.3	18.4	Tap et al. (2017)
Doxorubicin 75 mg/m^2^/d1/3w plus palifosfamide 150 mg/m^2^/d1–3/3w	2016	Phase III trial	First-line	226	28.3	6.0	15.9	Ryan et al. (2016)
Doxorubicin 25 mg/m^2^/d1–3/3w plus ifosfamide 2.5 g/m^2^/d1–4/3w	2014	Phase III trial	First-line	228	26	7.4	14.3	Judson et al. (2014)
Doxorubicin 30 mg/m^2^/d1–3/2w for 3 cycles followed by ifosfamide 2.5 g/m^2^/d1–5/3w for 3 cycles	2009	Phase II trial	First-line	62	24.1	6.0	-	Maurel et al. (2009)
Doxorubicin 20 mg/m^2^/d1–3/3w plus ifosfamide 2.5 g/m^2^/d1–3/3w plus dacarbazine 300 mg/m^2^/d1–3/3w	2009	Phase III trial	First-line	74	35	9.8	17.7	Fayette et al. (2009)
Doxorubicin 25 mg/m^2^/d1–3/3w plus ifosfamide 3 g/m^2^/d1–3/3w plus dacarbazine 400 mg/m^2^/d1–3/3w	2009	Phase III trial	First-line	71	38	9.1	17.3	Fayette et al. (2009)
Doxorubicin 30 mg/m^2^/d1–3/2w for 3 cycles followed by ifosfamide 2.5 g/m^2^/d1–5/3w for 3 cycles	2004	Phase II trial	First-line	57	38	5.6	13.5	Maurel et al. (2004)
Ifosfamide 2 g/m^2^/d1–2/3w plus etoposide 100 mg/m^2^/d1–5/3w plus cisplatin 20 mg/m^2^/d1–5/3w	2000	Phase II trial	First-line	104	46	4.6	8	Pápai et al. (2000)
Doxorubicin 50 mg/m^2^/d1/3w plus ifosfamide 5 g/m^2^/d1/3w	2000	Phase III trial	First-line	149	21	11.0	13.1	Le Cesne et al. (2000)
Doxorubicin 75 mg/m^2^/d1/3w plus ifosfamide 5 g/m^2^/d1/3w	2000	Phase III trial	First-line	145	23.3	8.6	12.8	Le Cesne et al. (2000)
Ifosfamide 1.5 g/m^2^/d1–3/3w plus etoposide 600 mg/m^2^/d1/3w	1997	Phase II trial	First-line	86	41	-	19	Saeter et al. (1997)
Doxorubicin 50 mg/m^2^/d1/3w plus ifosfamide 5 g/m^2^/d1/3w	1995	Phase III trial	First-line	258	28.1	10.3	12.8	Santoro et al. (1995)
Cyclophosphamide 500 mg/m^2^/d1/3w plus vincristine 1.5 mg/m^2^/d1/3w plus doxorubicin 50 mg/m^2^/d1/3w plus dacarbazine 750 mg/m^2^/d1/3w	1995	Phase III trial	First-line	142	28.4	11.2	11.9	Santoro et al. (1995)
Doxorubicin 60 mg/m^2^/d1/3w plus dacarbazine 1,000 mg/m^2^/d1/3w plus ifosfamide 5–7.5 g/m^2^/d1–3/3w	1993	Phase III trial	First-line	170	32	6	13	Antman et al. (1993)
Doxorubicin 60 mg/m^2^/d1/3w plus ifosfamide 5 g/m^2^/d1/3w	1989	Phase II trial	First-line	42	36	7	8	Loehrer et al. (1989)
Doxorubicin 60 mg/m^2^/d1/3w plus dacarbazine 900 mg/m^2^/d1/3w plus ifosfamide 7.5 g/m^2^/d1/3w	1989	Phase II trial	First-line	105	47	9.5	16	Elias et al. (1989)
Cyclophosphamide 200 mg/d1–7,15–21/4w plus sirolimus 4 mg/d	2012	Phase II trial	≥2 line	48	2	3.4	9.9	Schuetze et al. (2012)

STSs, soft tissue sarcomas; ORR, objective response rate; M-PFS, median progression-free survival; M-OS, median overall survival.

Among the chemotherapeutic drugs for STSs, ifosfamide is the second most effective after doxorubicin (Tascilar et al., 2007). Ifosfamide was first synthesized in the 1960s. It was introduced as a chemical modification of cyclophosphamide with a different position of its two chloroethyl groups on the central ring, providing a structure with greater water solubility and antitumor activity and a better toxicity profile (Kerbusch et al., 2001; Misiura, 2006; Tascilar et al., 2007). Numerous clinical trials and retrospective studies have demonstrated the efficacy of ifosfamide alone or ifosfamide-based chemotherapy for the treatment of STSs. The results of representative multicenter prospective clinical trials are presented in Table 2. Because there are no cardiotoxicity concerns, ifosfamide can be administered at significantly higher doses than doxorubicin. Current evidence indicates that the ORR of using large doses of ifosfamide to treat STSs is significantly higher than that of using low doses (Table 1). The efficacy of ifosfamide in the treatment of STSs is slightly lower than that of doxorubicin (ORR, 5.5%–25% vs. 5%–25%; median PFS, 2.2–3.5 vs. 2.5–10.7 months; median OS, 7.2–12.8 vs. 8.2–19.7 months, respectively) (Table 1; Table 2). Therefore, doxorubicin is still considered the first choice of chemotherapy for advanced STSs (Lorigan et al., 2007). However, ifosfamide may be superior to doxorubicin in synovial sarcoma (Nielsen et al., 2000b; Carter et al., 2020).

In the real world, ifosfamide is most commonly used in combination with doxorubicin (Table 1; Table 2). The ORR of the doxorubicin plus ifosfamide in treating STS is 21%–38%, and the median PFS is 5.6–11 months (Table 1; Table 2). Compared with doxorubicin or ifosfamide alone, the combination of doxorubicin and ifosfamide increases the ORR and median PFS but does not improve the median OS in patients with advanced STSs (Table 1; Table 2) (Maurel et al., 2009; Judson et al., 2014; Wang et al., 2021). Currently, this combined regimen is recommended for preoperative neoadjuvant chemotherapy in high-risk STSs (Judson et al., 2014; Weiss et al., 2020). Other drugs that are commonly used in combination with ifosfamide include dacarbazine and etoposide (Table 2). Notably, the doxorubicin plus ifosfamide plus dacarbazine has the highest ORR (32%–47%) and median PFS (6–9.8 months) in patients with advanced STSs (Table 2). The combination of these three drugs has not received sufficient attention in the era of targeted therapy and immunotherapy for STSs.

In summary, as a chemotherapeutic agent that is as well-known as doxorubicin, ifosfamide has an important effect on the chemotherapy of STSs. Ifosfamide is also worthy of further testing for the treatment of synovial sarcoma. However, the other oxazaphosphorines have not exceeded the role of ifosfamide in STSs.

### 2.3 Trabectedin

Trabectedin is a natural compound initially isolated from the marine ascidian *Ecteinascidia turbinata* and can be obtained by high-purity chemical synthesis (Trabectedin, 2003; Cuevas and Francesch, 2009; Ganjoo and Patel, 2009). It has a unique structure with three-fused tetrahydroisoquinoline rings, which allow it to inhibit cancer cells by causing single- and double-strand DNA breaks, and several other key cellular biological processes and tumor microenvironments (Trabectedin, 2003; Cuevas and Francesch, 2009; Gordon et al., 2016; Larsen et al., 2016; Ratan and Patel, 2017; Wang et al., 2022). Trabectedin was approved in Europe in 2007 for the treatment of advanced STSs with previous anthracycline treatment failure and in the United States in 2015 for the treatment of patients with advanced leiomyosarcoma and liposarcoma with previous anthracycline treatment failure (Nakamura and Sudo, 2022). It is the most studied and widely used chemotherapeutic drug for STSs, in addition to doxorubicin and ifosfamide (Rastogi and Bakhshi, 2016; Dang et al., 2021; Le Cesne, 2022; Nakamura and Sudo, 2022; Wang et al., 2022). The ORR of trabectedin monotherapy for STS is 4.7%–14.8%, the median PFS is 2.8–5.9 months, and the median OS is 9.2–21.3 months (Table 3). Although these data are similar to those of doxorubicin or ifosfamide monotherapy, recent randomized controlled studies have demonstrated that trabectedin cannot replace doxorubicin as a first-line treatment for advanced STSs (Bui-Nguyen et al., 2015; Martin-Broto et al., 2016). In addition, several studies have demonstrated that the efficacy of trabectedin in the treatment of leiomyosarcoma and liposarcoma at the second- or above-line setting is significantly higher than in other STS subtypes (median PFS 5.1 *versus* 1.4 months, respectively) (Rastogi and Bakhshi, 2016; Schuetze, 2021; Vincenzi et al., 2023). Therefore, it is necessary to conduct randomized controlled clinical trials in a first-line setting to compare the activity of trabectedin and doxorubicin in these histological subtypes (Blay et al., 2014; Dang et al., 2021).

**TABLE 3:** Outcomes of representative clinical trials of trabectedin in nonspecific STSs.

Drugs and dosages	Years of report	Study types	Setting	Number of patients	Outcomes			References
					ORR	M-PFS (months)	M-OS (months)	
Trabectedin 1.5 mg/m^2^/24 h–infusion/3w	2022	Phase IV trial	≥2 line	128	11.7%	5.2	15.2	Grunwald et al. (2022)
Trabectedin 1.5 mg/m^2^/24 h–infusion/3w	2021	Phase III trial	≥2 line	52	13.7%	3.1	13.6	Le Cesne et al. (2021)
Trabectedin 1.5 mg/m^2^/24 h–infusion/3w	2020	Phase II trial	First-line	24	-	4	12	Grosso et al. (2020)
Trabectedin 1.5 mg/m^2^/24 h–infusion/3w	2017	Phase IV trial	No line	218	26.6%	5.9	21.3	Buonadonna et al. (2017)
Trabectedin 1.2 mg/m^2^/24 h–infusion/3w	2015	Phase II trial	≥2 line for translocation-related sarcomas	37	8%	5.6	-	Kawai et al. (2015)
Trabectedin 1.3 mg/m^2^/3 h–infusion/3w	2015	Phase II trial	First-line	47	14.8%	2.8	-	Bui-Nguyen et al. (2015)
Trabectedin 1.5 mg/m^2^/24 h–infusion/3w	2015	Phase II trial	First-line	43	4.7%	3.1	-	Bui-Nguyen et al. (2015)
Trabectedin 1.5 mg/m^2^/24 h–infusion/3w	2014	Phase III trial	First-line for translocation-related sarcomas	61	5.9%	16.1	38.9	Blay et al. (2014)
Trabectedin 1.5 mg/m^2^/24 h–infusion/3w	2005	Phase II trial	≥2 line	99	8.1%	3.5	9.2	Le Cesne et al. (2005)
Trabectedin 1.1 mg/m^2^/d1/3w plus doxorubicin 60 mg/m^2^/d1/3w	2016	Phase II trial	First-Line	54	17%	5.7	13.3	Martin-Broto et al. (2016)

STSs, soft tissue sarcomas; ORR, objective response rate; M-PFS, median progression-free survival; M-OS, median overall survival.

### 2.4 Taxanes

Taxanes are an important class of antitumor drugs that can interfere with the function of microtubules in cells, leading to chromosomal non-aggregation in multipolar spindles, mitotic failure, and ultimately cell death induction (Yared and Tkaczuk, 2012; Weaver, 2014). They include paclitaxel and docetaxel and various analogs or processes thereof.

Paclitaxel was originally extracted from Pacific yew trees with a minimal yield. After its synthesis, paclitaxel has been widely used for the treatment of many cancers with significant therapeutic effects (Mekhail and Markman, 2002). However, the efficacy of paclitaxel monotherapy in the treatment of the majority of STSs is poor (Casper et al., 1998). Currently, paclitaxel alone is the recommended treatment for angiosarcoma (Skubitz and Haddad, 2005; Bui et al., 2018; Pink et al., 2021). A 2004 study demonstrated that a combination of paclitaxel and liposomal doxorubicin achieved appropriate efficacy in the treatment of STSs (Bafaloukos et al., 2004). However, because this chemotherapy regimen has no significant advantages over other regimens, it is rarely mentioned.

Docetaxel is a reprocessed taxol-like substance produced by the needles of *Taxus chinensis*. The chemical structures between docetaxel and paclitaxel differ in two ways (Ojima et al., 2016). These small changes make docetaxel different from paclitaxel in terms of water solubility, cellular effects, and pharmacology (Zhang et al., 2019). However, docetaxel monotherapy for STSs has also been proven ineffective (Santoro et al., 1999; Verweij et al., 2000). The efficacy of the combination of docetaxel and gemcitabine in the treatment of STSs is significantly higher than that of docetaxel alone or gemcitabine alone (Maki et al., 2007). Moreover, the efficacy of docetaxel plus gemcitabine is comparable to that of doxorubicin alone (Table 4). However, docetaxel plus gemcitabine is cumbersome, costly, and toxic than doxorubicin monotherapy; therefore, it is not recommended as a first-line treatment for advanced STSs (Seddon et al., 2017). Notably, docetaxel plus gemcitabine is deemed more effective in patients with leiomyosarcoma than in patients with other histological subtypes (Bay et al., 2006; Maki, 2007; Pautier et al., 2012; Choi et al., 2018).

**TABLE 4:** Outcomes of representative clinical trials of taxanes in nonspecific STSs.

Drugs and dosages	Years of report	Study types	Setting	Number of patients	Outcomes			References
					ORR (%)	M-PFS (months)	M-OS (months)	
**Single agent**								
Docetaxel 100 mg/m^2^/d1/3w	2001	Phase II trial	≥2 line	27	15	2.4	7.7	Köstler et al. (2001)
Docetaxel 100 mg/m^2^/d1/3w	2000	Phase II trial	First-line	42	0	1.6	9.8	Verweij et al. (2000)
Docetaxel 100 mg/m^2^/d1/3w	1999	Phase II trial	≥2 line	36	2.8	1.4	11.7	Santoro et al. (1999)
Docetaxel 100 mg/m^2^/d1/3w	1998	Phase II trial	First-line	30	10.7	-	-	Bramwell et al. (1998)
Docetaxel 100 mg/m^2^/d1/3w	1996	Phase II trial	First-line	18	5.9	-	-	Edmonson et al. (1996)
Paclitaxel 250 mg/m^2^/d1/3w	1998	Phase II trial	Any-line	28	7	3.5	-	Casper et al. (1998)
Paclitaxel 200 mg/m^2^/d1/3w	1997	Phase II trial	≥2 line	19	0	-	-	Patel et al. (1997)
Paclitaxel 250 mg/m^2^/d1/3w	1995	Phase II trial	First-line	48	12.5	1.6	12	Balcerzak et al. (1995)
**Combination regimens**								
Gemcitabine 900 mg/m^2^/d1,8/3w plus docetaxel 100 mg/m^2^/d8/3w	2021	Phase II trial	≥2 line	45	18	4.1	15.9	Somaiah et al. (2021)
Gemcitabine 900 mg/m^2^/d1,8/3w plus docetaxel 75 mg/m^2^/d8/3w	2019	Phase II trial	Any-line	70	20	5.6	21.1	Jones et al. (2019)
Gemcitabine 675 mg/m^2^/d1,8/3w plus docetaxel 75 mg/m^2^/d8/3w	2017	Phase III trial	First-line	128	20	5.5	15.7	Seddon et al. (2017)
Gemcitabine 1,000 mg/m^2^/d1,8/3w plus docetaxel 35 mg/m^2^/d1,8/3w	2012	Phase II trial	≥2 line	30	16.7	2.5	8.4	Lee et al. (2012)
Gemcitabine 900 mg/m^2^/d1,8/3w plus docetaxel 100 mg/m^2^/d8/3w	2007	Phase II trial	Any-line	73	16	6.2	17.9	Maki et al. (2007)
Pegylated liposomal doxorubicin 45 mg/m^2^/d1/4w plus paclitaxel 150 mg/m^2^/d1/4w	2004	Phase II trial	First-line	42	16	5.7	13.2	Bafaloukos et al. (2004)

STSs, soft tissue sarcomas; ORR, objective response rate; M-PFS, median progression-free survival; M-OS, median overall survival.

In summary, single-drug taxane is not recommended for the treatment of STSs. However, the docetaxel plus gemcitabine is considered second only to doxorubicin-based chemotherapy for STSs.

### 2.5 Gemcitabine

Gemcitabine is a cytotoxic nucleoside analog widely used in the treatment of malignant tumors. The metabolites of gemcitabine in cells can inhibit DNA synthesis via the inhibition of ribonucleotide reductase and compete with the nucleoside deoxycytidine as a fraudulent base, thereby producing antitumor effects (Barton-Burke, 1999; Wong et al., 2009). Although gemcitabine is widely used in other cancers, its efficacy alone in STSs is poor, with an ORR of 3%–8% and a median PFS of 1.5–3 months (Table 5). However, gemcitabine monotherapy has better efficacy in leiomyosarcoma and angiosarcoma (Pautier et al., 2012; Stacchiotti et al., 2012; Ducoulombier et al., 2016; Watson et al., 2022). Fortunately, gemcitabine combined with other drugs (such as docetaxel plus gemcitabine described above) can achieve better efficiency in STSs (Table 4; Table 5). Moreover, docetaxel plus gemcitabine has an efficacy comparable to that of doxorubicin-based chemotherapy in leiomyosarcoma and epithelioid sarcoma (Ducoulombier et al., 2016; Choi et al., 2018; Frezza et al., 2018). The efficacy of gemcitabine in combination with other drugs for STSs is inferior to that of docetaxel plus gemcitabine (Table 5). In addition, gemcitabine in combination with emerging drugs, such as pazopanib and eribulin, has also been tested for the treatment of STSs (Somaiah et al., 2021; Lopez-Alvarez et al., 2022). In summary, gemcitabine and docetaxel have similar efficacy and status in the treatment of STSs. The efficacy of their single-drug treatment is relatively low, whereas the gemcitabine plus docetaxel has comparable efficacy to first-line chemotherapy for STSs.

**TABLE 5:** Outcomes of representative clinical trials of gemcitabine in nonspecific STSs.

Drugs and dosages	Years of report	Study types	Setting	Number of patients	Outcomes			References
					ORR (%)	M-PFS (months)	M-OS (months)	
**Single agent**								
Gemcitabine 1,200 mg/m^2^/d1,8/3w	2007	Phase II trial	Any-line	49	8	3	11.5	Maki et al. (2007)
Gemcitabine 1,000 mg/m^2^/1w	2006	Phase II trial	First-line	46	7	2	6	Von Burton et al. (2006)
Gemcitabine 1,250 mg/m^2^/d1,8,15/4w	2003	Phase II trial	Any-line	25	4	-	15	Okuno et al. (2003)
Gemcitabine 1,250 mg/m^2^/d1,8/3w	2002	Phase II trial	≥2 line	31	3.23	1.5	8.9	Svancárová et al. (2002)
**Combination regimens**								
Gemcitabine 1,000 mg/m^2^/d1,8/3w plus pazopanib 800 mg/d	2021	Phase II trial	≥2 line	45	11	4.1	12.4	Somaiah et al. (2021)
Gemcitabine 900 mg/m^2^/d1,8/3w plus docetaxel 100 mg/m^2^/d8/3w	2021	Phase II trial	≥2 line	45	18	4.1	15.9	Somaiah et al. (2021)
Gemcitabine 1,000 mg/m^2^/d1,8/3w plus pazopanib 800 mg/d	2021	Phase II trial	≥2 line	43	11	5.6	13.1	Schmoll et al. (2021)
Gemcitabine 900 mg/m^2^/d1,8/3w plus docetaxel 75 mg/m^2^/d8/3w	2019	Phase II trial	Any-line	70	20	5.6	21.1	Jones et al. (2019)
Gemcitabine 675 mg/m^2^/d1,8/3w plus docetaxel 75 mg/m^2^/d8/3w	2017	Phase III trial	First-line	128	20	5.5	15.7	Seddon et al. (2017)
Gemcitabine 1,000 mg/m^2^/d1,8/3w plus docetaxel 35 mg/m^2^/d1,8/3w	2012	Phase II trial	≥2 line	30	16.7	2.5	8.4	Lee et al. (2012)
Gemcitabine 1800 mg/m^2^/3w plus dacarbazine 500 mg/m^2^/2w	2011	Phase II trial	≥2 line	57	12	4.2	16.8	Garcia-Del-Muro et al. (2011)
Gemcitabine 800 mg/m^2^/d1,8/3w plus vinorelbine 25 mg/m^2^/d1,8/3w	2007	Phase II trial	Any-line	40	10	3.4	-	Dileo et al. (2007)

STSs, soft tissue sarcomas; ORR, objective response rate; M-PFS, median progression-free survival; M-OS, median overall survival.

### 2.6 Dacarbazine

Dacarbazine is an alkylating agent, similar to oxazaphosphorine, which binds to DNA through metabolites in the body and establishes cross connections between the two strands, causing DNA replication to stop and ultimately leading to cell death (Huitema et al., 2000; Kantrowitz-Gordon et al., 2018; Karati et al., 2022). Additionally, dacarbazine exerts immune-stimulatory effects (Ugurel et al., 2013). Dacarbazine has a long history of application in STSs, with only mild activity, an ORR of 3%–4%, and a median PFS of 2–2.7 months (Table 6). Therefore, it is often used as a control drug in clinical trials of new drugs for second- or above-line treatment of STS (Demetri et al., 2016; Schoffski et al., 2016). In terms of combined use, dacarbazine is most commonly used in combination with doxorubicin and ifosfamide, and the efficacy is significant (Tables 1, 2, and 6). The combined regimen of dacarbazine and gemcitabine also has some efficacy in STSs (Table 6), but it is rarely used. In summary, as a veteran drug for the treatment of STSs, it is worthwhile to use dacarbazine in patients with STSs who have failed multiline treatment. In addition, a combined regimen of dacarbazine and other new drugs (such as trabectedin, eribulin, TKIs) is worth studying.

**TABLE 6:** Outcomes of representative clinical trials of dacarbazine in nonspecific STSs.

Drugs and dosages	Years of report	Study types	Setting	Number of patients	Outcomes			References
					ORR (%)	M-PFS (months)	M-OS (months)	
**Single agent**								
Dacarbazine 1,200 mg/m^2^/d1/3w	2021	Phase II trial	≥2 line	79	3	2.7	8	Van Tine et al. (2021)
Dacarbazine 1,200 mg/m^2^/d1/3w	2011	Phase II trial	≥2 line	52	4	2	8.2	Garcia-Del-Muro et al. (2011)
**Combination regimens**								
Gemcitabine 1800 mg/m^2^/d1/3w plus dacarbazine 500 mg/m^2^/d1/2w	2011	Phase II trial	≥2 line	57	12	4.2	16.8	Garcia-Del-Muro et al. (2011)
Doxorubicin 20 mg/m^2^/d1–3/3w plus ifosfamide 2.5 g/m^2^/d1–3/3w plus dacarbazine 300 mg/m^2^/d1–3/3w	2009	Phase III trial	First-line	74	35	9.8	17.7	Fayette et al. (2009)
Doxorubicin 25 mg/m^2^/d1–3/3w plus ifosfamide 3 g/m^2^/d1–3/3w plus dacarbazine 400 mg/m^2^/d1–3/3w	2009	Phase III trial	First-line	71	38	9.1	17.3	Fayette et al. (2009)
Dacarbazine 500 mg/m^2^/d1/2w plus gemcitabine 1800 mg/m^2^/d1/2w	2007	Phase II trial	≥2 line	23	4	3.6	8.6	Losa et al. (2007)
Cyclophosphamide 500 mg/m^2^/d1/3w plus vincristine 1.5 mg/m^2^/d1/3w plus doxorubicin 50 mg/m^2^/d1/3w plus dacarbazine 750 mg/m^2^/d1/3w	1995	Phase III trial	First-line	142	28.4	11.2	11.9	Santoro et al. (1995)
Doxorubicin 60 mg/m^2^/d1/3w plus dacarbazine 1,000 mg/m^2^/d1/3w	1993	Phase III trial	First-line	170	17	4	12	Antman et al. (1993)
Doxorubicin 60 mg/m^2^/d1/3w plus dacarbazine 1,000 mg/m^2^/d1/3w plus ifosfamide 5–7.5 g/m^2^/d1–3/3w	1993	Phase III trial	First-line	170	32	6	13	Antman et al. (1993)
Doxorubicin 60 mg/m^2^/d1/3w plus dacarbazine 900 mg/m^2^ plus ifosfamide 7.5 g/m^2^/d1/3w	1989	Phase II trial	First-line	105	47	9.5	16	Elias et al. (1989)
Doxorubicin 60 mg/m^2^/d1/3w plus dacarbazine 250 mg/m^2^/d1/3w	1987	Phase III trial	First-line	104	33	7.2	8.6	Baker et al. (1987)
Doxorubicin 60 mg/m^2^/d1/3w plus dacarbazine 250 mg/m^2^/d1/3w plus cyclophosphamide 500 mg/m^2^/d1/3w	1987	Phase III trial	First-line	112	34	6.0	9.8	Baker et al. (1987)
Doxorubicin 60 mg/m^2^/d1/3w plus dacarbazine 250 mg/m^2^/d1/3w plus actinomycin D 1.2 mg/m^2^/d1/3w	1987	Phase III trial	First-line	119	24	5.4	11.7	Baker et al. (1987)

STSs, soft tissue sarcomas; ORR, objective response rate; M-PFS, median progression-free survival; M-OS, median overall survival.

### 2.7 Eribulin

Similar to taxanes, eribulin inhibits microtubule polymerization. Similar to trabectedin, it is an anticancer drug found in marine organisms (Ratan and Patel, 2017). Eribulin is a synthetic analog of the naturally occurring anticancer agent halichondrin B in marine sponges (Shetty and Gupta, 2014). It exerts anticancer effects via multiple pathways. These pathways include the normalization of tumor blood vessels, inhibition of microtubule growth, isolation of microtubule proteins, reduction of microtubule supply, and reversal of the transition from mesenchymal to epithelial cells (Young and Woll, 2016; Phillips et al., 2022). In addition, eribulin has an important effect on the tumor immune microenvironment (Phillips et al., 2022). Although eribulin has various antitumor mechanisms, single-drug chemotherapy has limited efficacy in STSs (ORR, 0%–8%; median PFS, 2–4 months) (Table 7). However, eribulin alone has better efficacy in leiomyosarcoma and liposarcoma, especially in liposarcoma (Kawai et al., 2022). Owing to the short time since eribulin was approved for the treatment of STSs, there have been no clinical trials on eribulin-based combined chemotherapy for STSs. Eribulin also has therapeutic effects on angiosarcoma, pleomorphic sarcoma, synovial sarcoma, rhabdomyosarcomas, and myxofibrosarcoma (Phillips et al., 2022). Therefore, it is necessary to continue studying the activity of various eribulin-based combination regimens in STSs.

**TABLE 7:** Outcomes of representative clinical trials of eribulin and other drugs in nonspecific STSs.

Drugs and dosages	Years of report	Study types	Setting	Number of patients	Outcomes			References
					ORR (%)	M-PFS (months)	M-OS (months)	
Eribulin 1.4 mg/m2/d1,8/3w	2022	Phase IV trial	Any-line	252	8.1	2.5	10.8	Kawai et al. (2022)
Eribulin 1.4 mg/m^2^/d1,8/3w	2017	Phase II trial	≥2 line	51	0	4.1	13.2	Kawai et al. (2017)
Eribulin 1.4 mg/m^2^/d1,8/3w	2011	Phase II trial	≥2 line	115	4.3	2.1/2.9^a^	-	Schoffski et al. (2011)
Pemetrexed 500 mg/m^2^/d1/3w plus cisplatin 75 mg/m^2^/d1/3w	2021	Phase II trial	≥2 line	37	13.5	2.6	52	Kim et al. (2021)
Pemetrexed 500 mg/m^2^/d1/3w	2013	Phase II trial	≥2 line	48	5	1.6	6	Hartmann et al. (2013)
Ifosfamide 2 g/m^2^/d1–2/3w plus etoposide 100 mg/m^2^/d1–5/3w plus cisplatin 20 mg/m^2^/d1–5/3w	2000	Phase II trial	First-line	104	46	4.6	8	Pápai et al. (2000)
Ifosfamide 1.5 g/m^2^/d1–3/3w plus etoposide 600 mg/m^2^/d1/3w	1997	Phase II trial	First-line	86	41	-	19	Saeter et al. (1997)
Cisplatin 100 mg/m^2^/d1/3w plus vinblastine 1.2 mg/m^2^/d1/3w	1997	Phase II trial	≥2 line	20	0	-	-	Keohan et al. (1997)
Etoposide 50 mg/m^2^/d1–21/4w	1997	Phase II trial	≥2 line	27	0	-	-	Keizer et al. (1997)
Epirubicin 60 mg/m^2^/d1–3/3w plus cisplatin 30 mg/m^2^/d2–5/3w	1997	Phase II trial	First-line	56	54	-	-	Jelić et al. (1997)
Etoposide 200 mg/m^2^/72 h–infusion/3w	1997	Phase II trial	≥2 line	16	0	1.4	3.7	Crawley et al. (1997)
Cyclophosphamide 500 mg/m^2^/d1/3w plus vincristine 1.5 mg/m^2^/d1/3w plus doxorubicin 50 mg/m^2^/d1/3w plus dacarbazine 750 mg/m^2^/d1/3w	1995	Phase II trial	First-line	142	28.4	11.2	11.9	Santoro et al. (1995)
Cisplatin 400 mg/m^2^/d1–5/4w	1990	Phase II trial	Any-line	40	15	-	-	Budd et al. (1990)
Epirubicin 60 mg/m^2^/d1–3/3w plus cisplatin 30 mg/m^2^/d2–5/3w	1990	Phase II trial	First-line	35	57	-	-	Jelić et al. (1990)
Doxorubicin 70 mg/m^2^/d1/3w plus vindesine 3 mg/m^2^/d1/3w	1990	Phase III trial	First-line	147	18	4	9.9	Borden et al. (1990)
Cisplatin 120 mg/m^2^/3w	1987	Phase II trial	≥2 line	26	4	-	-	Sordillo et al. (1987)
Cisplatin 120 mg/m^2^/3w	1982	Phase II trial	≥2 line	36	6	-	-	Brenner et al. (1982)
Methotrexate 4 g/m^2^/3w	1980	Phase II trial	≥2 line	18	5.6	-	-	Karakousis et al. (1980)

STSs, soft tissue sarcomas; ORR, objective response rate; M-PFS, median progression-free survival; M-OS, median overall survival.

^a^ M-PFS, was 2.6, 2.9, 2.6, 2.1 months in the patients with adipocytic sarcoma, leiomyosarcoma, synovial sarcoma, and other types of STS, respectively.

### 2.8 Other chemotherapeutic drugs

In addition to the abovementioned drugs for treating STSs, many other drugs have been tested for the treatment of STSs. Vinca alkaloids (vindesine, vinblastine, vinorelbine, vincristine) have been widely tested in STSs, ultimately proving that they have important therapeutic effects in specific histological subtypes of STSs, such as rhabdomyosarcomas, whereas their activity in other subtypes is weak (Table 7). Methotrexate is one of the main drugs for the treatment of osteosarcomas (Belayneh et al., 2021). However, it is not involved in STS activity (Table 7) (Karakousis et al., 1980). Similarly, cisplatin is also one of the main drugs used for the treatment of osteosarcomas (Belayneh et al., 2021), with only slight activity in STSs (Table 7) (Brenner et al., 1982; Sordillo et al., 1987; Budd et al., 1990). The combined regimen of cisplatin with vinblastine or pemetrexed shows poor efficacy in STSs (Table 7) (Keohan et al., 1997; Kim et al., 2021). Cisplatin plus epirubicin has some activity in STSs (Table 7) (Jelić et al., 1990; Jelić et al., 1997), but this regimen is rarely used in the real world due to its high toxicity (Leahy et al., 2012; Nagar et al., 2018; Kim et al., 2019). As a widely used anticancer drug, etoposide has been tested repeatedly in STSs (Belani et al., 1994). However, whether administered orally or intravenously, the activity of etoposide alone in the STS is weak (Table 7) (Licht et al., 1994; Crawley et al., 1997; Keizer et al., 1997; Kebudi et al., 2004). Although ifosfamide plus etoposide shows some activity in STSs (Table 7) (Saeter et al., 1997; Yalçin et al., 1998; Pápai et al., 2000), this combined regimen is rarely used in the real world (Leahy et al., 2012; Nagar et al., 2018; Kim et al., 2019). In addition, researchers tested the activity of pemetrexed in STSs, and the results were disappointing (Table 7) (Hartmann et al., 2013; Kim et al., 2021).

In summary, anthracyclines, ifosfamide, trabectedin, gemcitabine, taxanes, dacarbazine and eribulin have certain activities in STSs. Vinca alkaloid agents (vindesine, vinblastine, vinorelbine, vincristine) have important therapeutic effects in specific STS subtypes, such as rhabdomyosarcomas, whereas their activity in other histological subtypes is weak. Other chemotherapeutic drugs (methotrexate, cisplatin, etoposide, pemetrexed) have weak efficacy in STSs and are rarely used.

## 3 Efficacy of different drugs in different STS histological subtypes

The high heterogeneity of STSs leads to a wide variety of histological subtypes. The rarity of STSs limits the development of large-scale, histologically specific clinical trials. Owing to the differences in the histological subtypes of STSs enrolled in clinical trials, there are differences in the efficacy of the same drug in various clinical trials. In addition, different STS subtypes respond differently to the same drugs. Therefore, the results of most clinical trials on advanced STSs are not applicable to all histological subtypes. To accurately describe the sensitivity of each STS subtype to different chemotherapeutic drugs, we analyzed the results of the prospective, multicenter clinical trials mentioned earlier in this review and recorded the efficacy of various drugs in different STS subtypes. Because most studies have not reported the remission results for each STS subtype in detail, the available results for some STS subtypes are sparse and limited. Therefore, we supplemented the results of some multicenter retrospective studies on some subtypes.

### 3.1 Leiomyosarcoma

Leiomyosarcoma can be divided into those with uterine and non-uterine sources. The clinical characteristics of the two types of leiomyosarcoma are slightly different, and currently, the treatment options for leiomyosarcoma from both sources are the same (Pautier et al., 2022). Leiomyosarcoma is the most prevalent STS histotype, with an incidence of 0.5–1/100,000 (Hung et al., 2019; Kim et al., 2019; Gronchi et al., 2020). Thus, there has been a significant inclusion of leiomyosarcoma in most trials of STSs. According to the obtained data, the most effective agent for single-drug chemotherapy of leiomyosarcoma is doxorubicin, with an ORR of 13%–22.6% and a median PFS of 6.2–6.9 months (Table 8) (Tap et al., 2020; Pautier et al., 2022). The second is trabectedin or gemcitabine alone, which can also result in a median PFS of >4 months (Table 8) (Pautier et al., 2012; Demetri et al., 2016; Gadducci et al., 2018). Ifosfamide, dacarbazine, and eribulin alone show mild efficacy against leiomyosarcoma (Table 8). Currently, doxorubicin plus trabectedin is the most effective chemotherapy regimen for the treatment of leiomyosarcoma, with an ORR >36% and a median PFS of >12 months (Pautier et al., 2015). Gemcitabine-based combination chemotherapy also results in a median PFS of >6 months (Table 8). In addition, a retrospective study suggested that doxorubicin plus dacarbazine and doxorubicin plus ifosfamide also achieved better efficacy in advanced leiomyosarcoma (D'Ambrosio et al., 2020).

**TABLE 8:** Representative studies related to chemotherapy for advanced leiomyosarcoma.

Drugs	Years of report	Study types	Setting	Number of patients	ORR	M-PFS (months)	References
**Single agents**							
Doxorubicin 75 mg/m^2^/d1/3w	2022	Phase III trial	First-line	76	13%	6.2	Pautier et al. (2022)
Eribulin 1.4 mg/m^2^/d1,8/3w	2022	Phase IV trial	≥2 line	73	7%	2.8	Kawai et al. (2022)
Doxorubicin 75 mg/m^2^/3w	2020	Phase III trial	First-line	115	22.6%	6.9	Tap et al. (2020)
Trabectedin 1.5 mg/m^2^/d1/3w	2019	Phase III trial	≥2 line	282	10%	4.3	Patel et al. (2019)
Dacarbazine 1,000 mg/m^2^/d1/3w	2019	Phase III trial	≥2 line	141	7%	1.6	Patel et al. (2019)
Trabectedin 1.3 mg/m^2^/3w	2018	Phase II trial	≥2 line	126	23.5%	4.1	Gadducci et al. (2018)
Trabectedin 1.5 mg/m^2^/d1/3w	2016	Phase III trial	≥2 line	152	-	4.3	Demetri et al. (2016)
Dacarbazine 1,000 mg/m^2^/d1/3w	2016	Phase III trial	≥2 line	126	-	1.6	Demetri et al. (2016)
Gemcitabine 1,000 mg/m^2^/d1,8,15/4w	2012	Phase II trial	≥2 line	43	16%	5.5/6.3^a^	Pautier et al. (2012)
Eribulin 1.4 mg/m^2^/d1,8/3w	2011	Phase II trial	≥2 line	40	5%	2.9	Schoffski et al. (2011)
Eribulin 1.5 mg/m^2^/d1/3w	2005	Phase II trial	≥2 line	43	11.6%	-	Le Cesne et al. (2005)
Ifosfamide 12 g/m^2^/3d–infusion/4w	2000	Phase II trial	Any-line	38	5%	-	Nielsen et al. (2000b)
**Combination regimens**							
Doxorubicin 60 mg/m^2^/d1/3w plus trabectedin 1.1 mg/m^2^/d1/3w	2022	Phase III trial	First-line	74	36%	12.2	Pautier et al. (2022)
Pemetrexed 500 mg/m^2^/d1/3w + cisplatin 75 mg/m^2^/d1/3w	2021	Phase III trial	≥2 line	10	0%	-	Kim et al. (2021)
Doxorubicin 75 mg/m^2^/d1/3w plus olaratumab 20 mg/kg/d1,8/3w in cycle 1 and 15 mg/kg in subsequent cycles	2020	Phase III trial	First-line	119	13.4%	4.3	Tap et al. (2020)
Gemcitabine 100 mg/m^2^/d1,8/3w plus pazopanib 800 mg/d	2020	Phase II trial	≥2 line	106	23.8%	6.5	Pautier et al. (2020)
Doxorubicin plus dacarbazine	2020	Retrospective	First-line	107	30.9%	9.2	D'Ambrosio et al. (2020)
Doxorubicin plus ifosfamide	2020	Retrospective	First-line	71	19.5%	8.2	D'Ambrosio et al. (2020)
Doxorubicin	2020	Retrospective	First-line	115	25.6%	4.8	D'Ambrosio et al. (2020)
Gemcitabine 900 mg/m^2^/d1,8/3w plus docetaxel 75 mg/m^2^/d8/3w	2018	Phase II trial	≥2 line	42	29%	6.9	Gadducci et al. (2018)
Doxorubicin 60 mg/m^2^/d1//3w plus trabectedin 1.1 mg/m^2^/d1/3w	2015	Phase II trial	First-line	108	48%	8.2/12.9^b^	Pautier et al. (2015)
Gemcitabine 900 mg/m^2^/d1,8/3w plus docetaxel 100 mg/m^2^/d8/3w	2012	Phase II trial	≥2 line	40	15%	3.8/4.7^c^	Pautier et al. (2012)
Doxorubicin 50–75 mg/m^2^/d1/3w plus ifosfamide 5 g/m^2^/d1/3w	2000	Phase III trial	First-line	112	14%	-	Le Cesne et al. (2000)

ORR, objective response rate; M-PFS, median progression-free survival.

^a^ M-PFS, was 5.5, 6.3 months in the patients with uterine leiomyosarcoma and nonuterine leiomyosarcoma, respectively.

^b^ M-PFS, was 8.2, 12.9 months in the patients with uterine leiomyosarcoma and nonuterine leiomyosarcoma, respectively.

^c^ M-PFS, was 4.7, 3.8 months in the patients with uterine leiomyosarcoma and nonuterine leiomyosarcoma, respectively.

In summary, doxorubicin-based chemotherapy remains the first-line treatment for advanced leiomyosarcoma, with doxorubicin plus trabectedin achieving the longest median PFS. Gemcitabine-based chemotherapy also has good efficacy in leiomyosarcoma. However, other chemotherapeutic drugs show lower activity against leiomyosarcoma.

### 3.2 Liposarcomas

Liposarcomas are divided into well differentiated, dedifferentiated, myxoid, round cell, and pleomorphic subtypes. Each histological subtype has a unique clinical presentation and therapeutic response (Lee et al., 2018). Therefore, differences in the histological subtypes of the recruited patients may lead to large differences in the outcomes of different clinical trials. However, owing to the rarity of various histological subtypes of liposarcoma, most clinical trials have not differentiated responses between different subtypes of liposarcoma. To date, the most effective chemotherapeutic drug for treating advanced liposarcomas is doxorubicin, with a median PFS of 6.7 months (Table 9) (Demetri et al., 2012). Gemcitabine plus docetaxel also shows good efficacy, with a median PFS of 5.6 months (Table 9) (Jones et al., 2019). Trabectedin and eribulin, which have high activity in liposarcoma, achieve a median PFS of only approximately 3 months in clinical trials related to liposarcoma (Table 9), which is significantly lower than that of doxorubicin-based chemotherapy. This may be related to the fact that almost all clinical trials of trabectedin and eribulin in liposarcoma are set at second- or above-line setting. In addition, dacarbazine can only achieve a median PFS of <2 months in liposarcoma (Table 9).

**TABLE 9:** Representative studies related to chemotherapy for advanced liposarcoma.

Drugs	Years of report	Study types	Setting	Number of patients	ORR	M-PFS (months)	References
Cabazitaxel 25 mg/m^2^/3w	2022	Phase II trial	1–2 line	38	8%	6	Sanfilippo et al. (2022)
Eribulin 1.4 mg/m^2^/d1,8/3w	2022	Phase IV trial	≥2 line	64	4.7%	3.2	Kawai et al. (2022)
Trabectedin 1.5 mg/m^2^/d1/3w	2019	Phase III trial	≥2 line	102	9%	3	Patel et al. (2019)
Dacarbazine 1,000 mg/m^2^/d1/3w	2019	Phase III trial	≥2 line	52	6%	1.5	Patel et al. (2019)
Gemcitabine 900 mg/m^2^/d1,8/3w plus docetaxel 75 mg/m^2^/d8/3w	2019	Phase II trial	Any-line	15	-	5.6	Jones et al. (2019)
Eribulin 1.4 mg/m2/d1,8/3w	2017	Phase III trial	≥3 line	71	1.4%	2.9	Demetri et al. (2017)
Dacarbazine 850–1,200 mg/m^2^/d1/3w	2017	Phase III trial	≥2 line	72	0%	1.7	Demetri et al. (2017)
Doxorubicin 75 mg/m^2^/d1/3w	2012	Phase II trial	First-line	22	-	6.7	Demetri et al. (2012)
Eribulin 1.4 mg/m^2^/d1,8/3w	2011	Phase II trial	≥2 line	37	3%	2.6	Schoffski et al. (2011)

ORR, objective response rate; M-PFS, median progression-free survival.

In summary, doxorubicin-based chemotherapy or gemcitabine plus docetaxel is the first recommended option for advanced liposarcomas. In addition, it is worth testing the activity of trabectedin or eribulin alone or in combination with other drugs for advanced liposarcomas in a first-line setting.

### 3.3 Synovial sarcoma

Synovial sarcoma is a rare histotype of STSs, with an incidence of 0.1–0.5/100,000 (Wibmer et al., 2010; Hung et al., 2019; Aytekin et al., 2020). Clinical trials specifically targeting synovial sarcoma are rare. Extracting detailed treatment data for patients with synovial sarcoma from most clinical trials of STSs is also difficult. Nevertheless, important information can still be obtained. In prospective clinical trials, the currently proven drug with the best efficacy for the treatment of synovial sarcoma is ifosfamide (Table 10) (Nielsen et al., 2000b; Tap et al., 2017). Doxorubicin and eribulin also exhibit certain activities in synovial sarcoma (Table 10). However, gemcitabine, docetaxel, and dacarbazine show only weak activity against synovial sarcoma (Table 10). Retrospective studies confirmed these conclusions (Ferrari et al., 2015; Sanfilippo et al., 2015; Desar et al., 2018; Pender et al., 2018; Stacchiotti and Van Tine, 2018; Carter et al., 2020; Kogushi et al., 2020). A retrospective study demonstrated that trabectedin had activity in synovial sarcoma (Sanfilippo et al., 2015).

**TABLE 10:** Representative studies related to chemotherapy for advanced synovial sarcomas.

Drugs	Years of report	Study types	Phase	Number of patients	ORR (%)	M-PFS (months)	References
Eribulin 1.4 mg/m^2^/d1,8/3w	2022	Phase IV trial	≥2 line	13	23	3.7	Kawai et al. (2022)
Eribulin 1.4 mg/m^2^/d1,8/3w	2011	Phase II trial	≥2 line	19	5	2.6	Schoffski et al. (2011)
Gemcitabine plus docetaxel	2007	Phase II trial	Any-line	9	0	-	Maki et al. (2007)
Doxorubicin 30 mg/m^2^/d1–2/3w plus ifosfamide 3.75 g/m^2^/d1–2/3w	2003	Phase II trial	First-line	12	42	-	Edmonson et al. (2003)
Ifosfamide 12 g/m^2^/3d–infusion/4w	2000	Phase II trial	Any-line	22	36	-	Nielsen et al. (2000b)
Doxorubicin plus ifosfamide	2000	Phase III trial	First-line	29	28	-	Le Cesne et al. (2000)
Doxorubicin plus dacarbazine	1991	Phase III trial	Any-line	8	0	-	Zalupski et al. (1991)

ORR, objective response rate; M-PFS, median progression-free survival.

### 3.4 Other STS histological subtypes

The ORR of doxorubicin plus ifosfamide for undifferentiated pleomorphic sarcoma (UPS) is 29% (Le Cesne et al., 2000), that of doxorubicin plus dacarbazine is 26% (Zalupski et al., 1991), that of gemcitabine plus docetaxel is 11%–36% (Maki et al., 2007; Choi et al., 2018; Jones et al., 2019), and that of eribulin is 11% (Kawai et al., 2022).

Clinical trials of chemotherapy for angiosarcoma are rare. The only clinical trial has demonstrated an ORR of 45% for paclitaxel plus bevacizumab for the treatment of advanced angiosarcoma (Bui et al., 2018). Numerous other retrospective studies have demonstrated that doxorubicin- and gemcitabine-based chemotherapies can also achieve efficacy similar to that of paclitaxel in angiosarcoma (Skubitz and Haddad, 2005; Schlemmer et al., 2008; Penel et al., 2012; Stacchiotti et al., 2012; Young et al., 2014; Choi et al., 2018; Watson et al., 2022). In addition, eribulin is believed to exert activity in angiosarcoma (Kawai et al., 2022).

Doxorubicin plus ifosfamide is the most effective chemotherapy for treating malignant peripheral nerve tumors (MPNTs) (with an ORR of >20%) (Kroep et al., 2011). Doxorubicin and dacarbazine also have therapeutic effects (Zalupski et al., 1991). Gemcitabine, docetaxel, and eribulin are also ineffective against MPNT (Maki et al., 2007; Choi et al., 2018; Kawai et al., 2022).

Doxorubicin plus ifosfamide has a similar efficacy to gemcitabine plus docetaxel in epithelioid sarcoma, and both have moderate activity (Choi et al., 2018; Frezza et al., 2018; Touati et al., 2018).

Gemcitabine plus docetaxel chemotherapy has mild activity in clear cell sarcoma (Cojocaru et al., 2020).

### 3.5 Specific STS histological subtypes

In terms of chemotherapy, the main specific STS histological subtypes include the rhabdomyosarcoma family and the Ewing sarcoma family of tumors (Granowetter et al., 2009; Gallego et al., 2021; Agaram, 2022). They are more sensitive to chemotherapeutic drugs than other STSs (Chen et al., 2019; Bisogno and Hawkins, 2020; Gallego et al., 2021; Riggi et al., 2021; Setty et al., 2023). However, the sensitive drugs of these specific STS histological subtypes are significantly different from non-specific STS subtypes. Rhabdomyosarcomas can be divided into several subtypes, and the first-line chemotherapy drugs varies among different subtypes (Agaram, 2022; Sparber-Sauer, 2022; Bisogno et al., 2023). The first-line chemotherapy drug for pleomorphic rhabdomyosarcoma and adult spindle cell rhabdomyosarcoma is usually doxorubicin (Gallego et al., 2021; Gronchi et al., 2021), and for other subtypes include ifosfamide, vincristine, actinomycin D, doxorubicin, cyclophosphamide, and vinorelbine (Walterhouse et al., 2014; Walterhouse et al., 2017; Bisogno et al., 2019; Schoot et al., 2022; Bisogno et al., 2023). Ewing sarcoma family of tumors are considered main members of small round cell sarcomas (Rajwanshi et al., 2009; Marino-Enriquez and Fletcher, 2014; Domanski, 2022; Gajdzis et al., 2022). Vincristine, doxorubicin, cyclophosphamide, ifosfamide, and etoposide are the first-line drugs recommended for Ewing sarcomas (Brennan et al., 2022). Vincristine, irinotecan, and temozolomide are the recommended drugs for patients with rhabdomyosarcoma or Ewing sarcoma with recurrent or frontline chemotherapy failure (Defachelles et al., 2021; Xu et al., 2023). Trabectedin, gemcitabine, taxanes, dacarbazine and eribulin show ineffective or uncertain efficacy against rhabdomyosarcomas or Ewing sarcomas (Etcubanas et al., 1985; Baruchel et al., 2012; Mora et al., 2017; Oesterheld et al., 2020; Kawai et al., 2022).

In addition to the STS subtypes described above, dozens of other STS subtypes require chemotherapy in advanced stages. However, no specific prospective clinical trial has confirmed the chemotherapeutic efficacy of these STS subtypes. Generally, most silent STS subtypes are treated based on data from nonspecific STS clinical trials (Tables 1–7).

## 4 Discussion

We conducted this review to provide a reference for the selection of chemotherapeutic drugs for advanced STSs. In this study, we comprehensively reviewed the results of representative clinical trials related to chemotherapy for STS over the past 30 years and supplemented with some retrospective studies. Numerous clinical trial results have shown that doxorubicin is the most effective drug and remains the mainstay of chemotherapy for advanced STSs. In addition, ifosfamide, trabectedin, gemcitabine, taxanes, dacarbazine, and eribulin have certain activities in STSs and are commonly used in the real world. Vinca alkaloid agents (vindesine, vinblastine, vinorelbine, vincristine) have important therapeutic effects in special STS subtypes, such as rhabdomyosarcomas and Ewing sarcomas, whereas their activity in other histological subtypes is weak. Other chemotherapeutic drugs (methotrexate, cisplatin, etoposide, pemetrexed) have weak efficacy against STSs and are rarely used. Sensitive chemotherapeutic drugs vary for each STS histotype. Doxorubicin-based chemotherapy is the most effective treatment for leiomyosarcoma, liposarcoma, UPS, angiosarcoma, MPNT, and epithelioid sarcoma. Ifosfamide is the most effective chemotherapeutic drug for synovial sarcoma. Gemcitabine plus docetaxel shows good efficacy against many STS subtypes. However, except for the few histological subtypes mentioned above, other STS subtypes have become the silent majority, and few large sample size studies have focused on and reported the chemotherapeutic efficacy of these STSs in detail.

With an increase in drugs used for second- or above-line treatment, a better understanding of histotype-oriented therapy, and improved supportive care in oncology, the survival period of patients with advanced STSs has increased over the past decade (Kollar et al., 2019; Smrke et al., 2020; Stricker et al., 2023). The number of treatment lines for these patients is increasing, as is the demand for sensitive chemotherapeutic drugs. This study has important reference value for drug selection in multiline therapy of STSs. In addition, with the widespread and in-depth application of targeted drugs in STSs, the selection of specific chemotherapeutic drugs based on different histological subtypes in combination with targeted drugs is inevitable to achieve better therapeutic effects. This study provides important reference value for the selection of chemotherapeutic drugs for these combined regimens.

STSs are characterized by a low incidence rate and high heterogeneity compared with other cancers. The low incidence rate has led to a considerable number of STS histological subtypes not being studied in depth and has also led to a delay in the research and development of chemotherapeutic drugs related to STSs. Almost no important chemotherapeutic drugs for STSs have emerged in the last decade. The high heterogeneity of STSs has led to significant differences in the outcomes of clinical trials of chemotherapy in different STSs, leading to an inability to accurately compare the efficacy of different drugs in STSs. For example, although many studies have confirmed that different histological subtypes of STSs have different sensitivities to chemotherapy, the results of an important clinical trial showed that in a population of patients with high-risk STSs, there was no benefit of neoadjuvant histotype-tailored chemotherapy regimens over the standard doxorubicin plus ifosfamide chemotherapy (Gronchi et al., 2017; Gronchi et al., 2020). To eliminate the influence of the low incidence rate and high heterogeneity of STSs on judging the efficacy of chemotherapeutic drugs, significant work needs to be carried out, including the following: 1) Clinical trials targeting different STS subtypes should be conducted as much as possible, whereas clinical trials targeting nonselective STSs should be conducted to reduce the effects of high heterogeneity. 2) Detailed histotype data should be reported for clinical trials of STSs. We found that many clinical trials did not report the histotype outcomes, leading to difficulties in histotype studies. Reporting detailed histotype data is a fundamental requirement for these clinical trials. 3) The evaluation criteria are unified. Early evaluation of the chemotherapeutic efficacy of STSs often uses ORR while ignoring other indicators. The ORR does not represent the median PFS and OS. Therefore, the reference value for early clinical trials is limited. Currently, the number of drugs and lines for advanced STSs has increased significantly, and there is a significant error in using the median OS as the main evaluation index. We recommend using the median PFS and 3- or 6-month PFS rates as the main evaluation indicators.

We conducted extensive searches and reviews to include all the relevant studies. However, our approach does not represent a complete review of the literature. For example, some studies may have been omitted because we only included studies published in English and excluded most of the retrospective studies. However, efforts have been made to ensure the inclusion of key studies on the treatment of advanced STSs.

In conclusion, anthracyclines are the most important systemic treatment for advanced STSs. Ifosfamide, trabectedin, gemcitabine, taxanes, dacarbazine, and eribulin exhibit certain activities in STSs. Other chemotherapeutic drugs have weak efficacy against STSs and are rarely used. Depending on the histological subtypes, it is necessary to select specific second- or above-line chemotherapeutic drugs.
